# Nutritional stress in larvae induces adaptive responses that transcend generations in males of a model insect

**DOI:** 10.1242/jeb.247972

**Published:** 2025-01-17

**Authors:** Lucy Rebecca Davies, Torsten N. Kristensen, Jesper G. Sørensen, Volker Loeschcke, Mads F. Schou

**Affiliations:** ^1^ Department of Biology, Aarhus University, DK-8000 Aarhus C, Denmark; ^2^ Department of Biological and Environmental Science, University of Jyväskylä, 40500 Jyväskylä, Finland; ^3^ Department of Chemistry and Bioscience, Aalborg University, DK-9220 Aalborg East, Denmark; ^4^ Department of Biology, Lund University, 223 62 Lund, Sweden

**Keywords:** Maternal effects, Starvation, *Drosophila*, Nutrition, Variable environment

## Abstract

The ability of organisms to cope with poor quality nutrition is essential for their persistence. For species with a short generation time, the nutritional environments can transcend generations, making it beneficial for adults to prime their offspring to particular diets. However, our understanding of adaptive generational responses, including those to diet quality, are still very limited. Here, we used the vinegar fly, *Drosophila melanogaster*, to investigate whether females developing as larvae on a nutritionally poor diet produce offspring that are primed for nutrient deficiencies in the following generations. We found that females developed on low-quality diets produced offspring that, on similarly low-quality diets, had both increased egg-to-adult viability and starvation tolerance compared with offspring of females experiencing a nutrient-rich diet. When testing the persistence of such generational priming, we found that just one generation of high-quality diet is sufficient to erase the signal of priming. A global transcriptomic profile analysis on male offspring suggests that the observed phenotypic priming is not a constitutive transcriptomic adjustment of adults; instead, offspring are probably primed as larvae, enabling them to initiate an adaptive response as adults when exposed to low-quality diets. Our results support that generational priming is an important adaptive mechanism that enables organisms to cope with transient nutritional fluctuations.

## Teaser

**Summary:** Adult *Drosophila* exposed to nutritional stress prime their offspring to survive in unfavourable environments. Priming is diluted after just one generation of optimal nutrition.

## INTRODUCTION

Most animals are exposed to fluctuating nutritional quality across generations, and even small changes in nutritional quality or quantity can impact their fitness (McAuley, 2022). When these effects also transcend to the life history and fitness traits of their offspring as generational or maternal effects (Uller, 2008; Nettle and Bateson, 2015), the importance for population performance and persistence is amplified (Sultan et al., 2009; Nettle and Bateson, 2015). Previous work on generational effects of nutrition has focused on how highly favourable or unfavourable environments restrict investment in offspring quality (Mousseau and Fox, 1998; Vijendravarma et al., 2010; Mautz et al., 2019). However, generational effects also offer an opportunity for species in predictable environments to prime their offspring to a particular environmental condition. For example, responses to circadian rhythms in three-spined sticklebacks (Magierecka et al., 2022), temperature in guppies (Le Roy and Seebacher, 2018) or social environment of mice (Nelson et al., 2013) can transcend generations, thereby improving offspring fitness in an environment matching that of the previous generation.

For species exposed to environmental fluctuations of high predictability across generations, the ability to prime offspring to cope with a poor environment would be highly beneficial. An example of this is from *Drosophila melanogaster*, where poor nutrition, especially during larvae development, has been shown to have sizeable negative effects on viability as well as adult body size and tolerance towards stressful environmental conditions (Tu and Tatar, 2003; McGraw et al., 2007; Kolss et al., 2009; Andersen et al., 2010; Rodrigues et al., 2015; Davies et al., 2018). It is therefore likely that selection favours the ability to prime offspring to a poor nutritional environment and reduce the negative impacts on fitness, if the priming comes without substantial costs. Previous work investigating nutritional priming has been limited by two challenges: (1) it requires multiple nutritional environments to ensure that the quality of nutrients is sufficiently low to detect a response, but not so stressful that it prevents an adaptive response, and (2) it requires multiple generations of testing to pinpoint the duration of priming before performance returns to initial levels. Identifying the duration (number of generations) of priming also serves to validate that the phenotypic change is not a permanent change caused by, for example, selection. Because of these challenges, there is currently no consensus in the literature as to whether or when generational effects of malnutrition can prime offspring for increased performance (Vijendravarma et al., 2010; Deas et al., 2019).

**Fig. 1.**
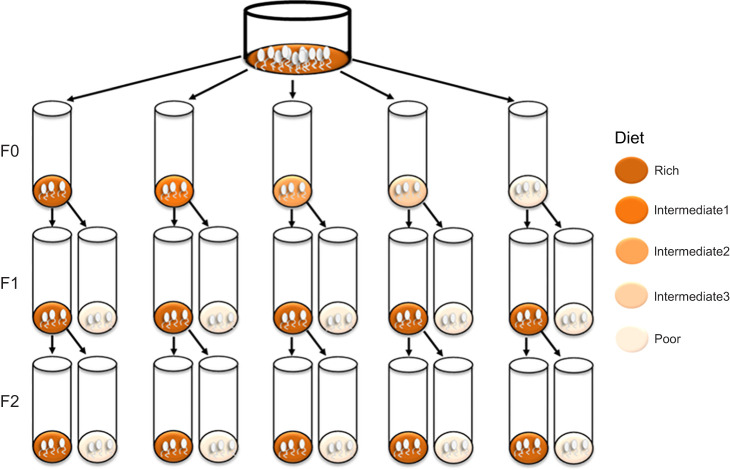
**Experimental design.** Flies were exposed to developmental nutrition treatments across three generations. In the first generation (F0), eggs of *Drosophila melanogaster* were distributed between vials containing different diets (nutritionally rich to poor, indicated by the increasing paleness of colours) and so developed over a range of nutritional stress. To test F1 flies for generational effects in their ability to cope with poor nutrition, we developed the F1 offspring on the Rich or Poor diet and assessed their egg-to-adult viability, and adult males were tested for starvation resistance. Finally, we tested the persistence of any generational effects for coping with poor nutrition by testing eggs and offspring of the F1 flies that had developed on the Rich F1 diet: F2 flies were also developed on the Rich or Poor diet and their egg-to-adult viability and the males' starvation tolerance were measured.

Here, we studied the generational effects of maternal and grandmaternal nutritional stress during development using the vinegar fly, *D. melanogaster*. Based on previous work (Schou et al., 2015), we designed a gradient of five developmental nutritional treatments ensuring exposure of flies to a range of stress levels (Table 1). We focused on maternal effects as these are likely to be more prominent in natural populations as females are generally more impacted by nutritional fluctuations than males (Harbison et al., 2005; Partridge et al., 2005; Dew-Budd et al., 2016; Duxbury and Chapman, 2020; Lushchak et al., 2023). When quantifying the maternal effects on subsequent generations, we focused on male progeny, where we expected less impact of mating and age on their sensitivity to nutritional conditions, compared with egg-producing female progeny (Partridge et al., 2005; Sieber and Spradling, 2015; Jang and Lee, 2015; Millington and Rideout, 2018; Wu et al., 2020). Female flies were exposed to a gradient in diet quality during their development, and generational effects in their offspring were quantified. First, we assessed egg-to-adult viability, and then the starvation resistance of male offspring was measured, for both Poor and Rich diets in the subsequent two generations (Fig. 1). Because previous work in mice has shown that restriction in maternal nutrition may affect gene expression in offspring generations (Lillycrop et al., 2005), we used global transcriptome profile analyses to investigate whether basal gene expression levels in males, before starvation (i.e. constitutive gene expression), were impacted by the maternal nutrition. Using this design, we tested: (1) whether females can prime their offspring to better cope with a low-quality feeding environment, (2) whether priming persists after exposure to improved nutritional environments, and (3) whether priming of offspring is associated with changes in the constitutive gene expression of adult males.

**Table 1.** Composition of the five developmental diets with different yeast concentrations

Diet	Yeast (g l^−1^)	Sugar (g l^−1^)	Oatmeal (g l^−1^)	Nipagin (ml l^−1^)	Acetic acid (ml l^−1^)	Agar (g l^−1^)
Rich	60	40	30	12	1.2	16
Intermediate1	21.1	0	0	12	1.2	16
Intermediate2	16.4	0	0	12	1.2	16
Intermediate3	11.6	0	0	12	1.2	16
Poor	6.8	0	0	12	1.2	16

The Rich diet is the in-house laboratory medium which, in addition to yeast, also contains oatmeal and sugar.

## MATERIALS AND METHODS

### Developmental diets

To expose flies to nutritional stress during development, we used four diets consisting of a progressive dilution of yeast from diet 1 (Intermediate1) to diet 4 (Poor) (Table 1). These diets were originally designed by Schou et al. (2015) to impose a stress in the nutritional environment of *D. melanogaster* larvae. In addition to these four diets, we also exposed flies to the in-house laboratory medium which in addition to yeast also contains oatmeal and sugar, and therefore represents a rich diet (Table 1).

### Fly stock

The population of *Drosophila melanogaster* Meigen 1830 used for this study was from a laboratory stock established from the offspring of approximately 600 inseminated females caught near Odder (55°56′42.46″N, 10°12′45.31″E), Denmark, in 2010 (Schou et al., 2014). From the time of establishment to the start of this experiment, flies were maintained on a 12 h:12 h light:dark cycle at 19°C on the Rich diet.

### Experimental flies

Before exposing flies to our range of developmental diets, the population went through two generations of acclimation. In these generations, flies were kept at a controlled density of 200 adult flies per plastic flask at 25°C on the Rich diet. This ensured that all experimental flies had the exact same grandmaternal and maternal environment and were acclimated to the same temperature. We started the experiment by establishing the F0 generation from eggs from the controlled and acclimated generation of flies. Specifically, eggs were distributed to the different developmental diets by transferring eggs to vials containing 7 ml of one of the five F0 diets at a density of 30 eggs per vial (Fig. 1). A total of 20 (19 for the Poor diet) vials were collected for each treatment, and the emerging flies were referred to as the F0 flies.

Next, we established the F1 generation by collecting eggs from the emerged F0 flies and transferring these to either the Rich diet or the most stressful diet, the Poor diet, during their development. The use of these two diets allowed us to test whether the expression of generational effects (in F0) was dependent on their nutritional environment (in F1). Eggs were obtained by housing virgin F0 females with control males: 10 females and 10 males in each of 10 vials on the Rich diet for 3 days. The control males were all developed on the Rich diet and were of the same age as the females. After 3 days of mating, the flies were transferred to new vials containing a spoon filled with agar and some dry yeast to optimise egg laying. Eggs were collected from the agar and placed in 20 (19 for F0-Intermediate1 to Poor and F0-Poor to Poor) vials of both the Rich and Poor diet, each with 30 eggs per vial (Fig. 1). The emerging flies are referred to as F1 flies.

Finally, to test whether any generational effects would persist after one additional generation at the Rich diet, we obtained offspring (F2) from the F1 flies developed on the Rich diet (Fig. 1). This was done following the same procedure as described above for the F0 generation.

### Measuring generational effects

To quantify generational effects in both F1 and F2, we measured egg-to-adult viability and adult starvation resistance. Egg-to-adult viability was estimated by counting the number of adults emerging from 30 eggs present in each replicate vial. Starvation resistance was assessed for males while females were used to create progeny for the next generation. Within 6 h of emergence from the developmental diets, flies were sexed by sight, and males were placed individually in vials containing 3.5 ml of a 2% agar solution. Every 6 h, we scored the number of dead flies until all flies had died. A total of 18–20 males was tested per treatment.

### Quantifying gene expression

To test whether the generational effects were detectable in gene expression, we focused on the flies from the two extreme treatments, as these showed the most significant differences in starvation resistance and egg-to-adult viability. We included F0 flies developed on the Rich and Poor diet, as well as their F1 and F2 progeny generations. We focused on the adult flies instead of larvae as it gave us better control of the age of individuals, reducing any unwanted variation in gene expression due to age differences among treatments. We collected 1 day old virgin males (*n*=15 per treatment) and flash froze them using liquid nitrogen. In each treatment, the 15 males were split into three replicates of five individuals.

#### Sample processing

RNA extraction and sample processing were performed at an Affymetrix service provider and core facility (KFB – Center of Excellence for Fluorescent Bioanalytics, Regensburg, Germany; www.kfb-regensburg.de). Full details of methods used by the service provider can be found here https://assets.thermofisher.com/TFS-Assets/LSG/manuals/MAN0017893_703262_GeneChipWT_Pico_UG.pdf.

#### Total RNA extraction

Total RNA was extracted according to the instructions in the RNeasy Plus Micro Kit (‘Purification of total RNA from animal and human cells’ protocol, Qiagen, Hilden, Germany). In brief, frozen whole flies were shipped on dry ice to KFB – Center of Excellence for Fluorescent Bioanalytics (www.kfb-regensburg.de). The flies were homogenised in 700 µl Trizol reagent using Precellys CK14 ceramic beads (2 cycles of 20 s at 6500 rpm; 15 s break). After 5 min incubation at room temperature, 140 µl of chloroform was added and the samples were again incubated at room temperature for 2 min. Phase separation was achieved by 15 min centrifugation at 12,000 ***g*** at 4°C. An equal amount of 70% ethanol was added to the aqueous supernatant and the mixture was applied to RNeasy Micro spin columns (RNeasy Micro Kit, Qiagen), followed by an on-column DNase digestion and several wash steps. Finally, total RNA was eluted in 14 μl of nuclease-free water. Purity and integrity of the RNA were assessed on an Agilent 2100 Bioanalyzer with the RNA 6000 Nano LabChip reagent set (Agilent, Palo Alto, CA, USA).

#### GeneChip microarray assay

Sample preparation for microarray hybridisation was carried out as described in the Affymetrix GeneChip WT Pico Reagent Kit User Guide (Affymetrix, Inc., Santa Clara, CA, USA). In brief, 5.0 ng of total RNA was used for reverse transcription to synthesise single-stranded cDNA with a T7 promoter sequence at the 5′ end. Next, a 3′ adaptor was added and the single-stranded cDNA was converted to double-stranded cDNA, which acted as a template for a pre-*in vitro* transcription (IVT) amplification by a low-cycle PCR (6 cycles). Afterwards, antisense RNA (complimentary RNA or cRNA) was synthesised and linearly amplified by IVT of the double-stranded cDNA template. Then, 20 µg of cRNA was purified and reverse transcribed into sense-strand cDNA, whereat unnatural dUTP residues were incorporated at a fixed ratio relative to dTTP. Purified sense-strand cDNA was fragmented using a combination of uracil DNA glycosylase (UDG) and apurinic/apyrimidinic endonuclease 1 (APE 1) at the dUTP residues followed by a terminal labelling with biotin. Next, 5.5 µg fragmented and labelled sense-strand cDNA was hybridised to Affymetrix Drosophila Gene 1.0 arrays for 16 h at 45°C in a GeneChip Hybridization Oven 640. Hybridised arrays were washed and stained in an Affymetrix Fluidics Station FS450, and the fluorescence signals were measured with an Affymetrix GeneChip Scanner 3000 7G. Fluidics and scan functions were controlled by the Affymetrix GeneChip Command Console v.4.1.3 software. Summarised probe set signals in log_2_ scale were calculated using the RMA algorithm (Irizarry et al., 2003) with the Affymetrix GeneChip Expression Console v.1.4 software.

### Statistical analysis

All statistical analyses were carried out in R version 4.1.3 (http://www.R-project.org/). Full statistical results can be found in Table S1.

#### Starvation resistance

We tested the effect of yeast concentration in F0 on starvation resistance (Gaussian, hours) using a linear model with yeast concentration (continuous) as a quadratic fixed effect using orthogonal polynomials. The statistical significance of yeast concentration in this model was estimated by step-wise reduction of the model from a quadratic effect of yeast concentration, to a linear effect of yeast concentration, and finally a null model. Model comparisons were done using *F*-tests. Results from the Rich diet in F0 are an important baseline and are therefore shown in all figures. However, the complex composition of the Rich diet (Table 1) puts it on a different scale from the media of varying yeast concentration, and it was therefore excluded from this and other analyses of F0 media.

To test for generational (i.e. maternal) effects in F1, we tested whether the performance of F1 flies on the Rich and Poor diets was dependent on the medium experienced by their parent generation. We therefore constructed linear models containing starvation resistance as the response variable and the fixed effects F0 diet (yeast concentration, continuous and quadratic) and F1 diets (Rich or Poor diet, factorial). We also included the interaction between F0 diet and F1 diet. We performed step-wise model reductions and comparisons using *F*-tests to evaluate: (1) whether the quadratic effect of yeast concentration in F0 was superior to the linear effect, by comparing a model with or without the quadratic effect; (2) using the superior model from 1 to test whether the F0 and F1 interaction was statistically significant, by comparing the model with an interaction to a model without the interaction. In the case of a significant interaction, we further evaluated the cause of this interaction by splitting the dataset by the two F1 diets (Rich and Poor diets) and testing the effect of the F0 developmental diet for each diet separately using *F*-tests. Finally, using the starvation resistance of F2 flies, we tested for grandmaternal effects using the same modelling strategy as for the maternal effects of F1 flies. All assumptions of the parametric analyses were fulfilled.

#### Egg-to-adult viability

To test for generational effects on egg-to-adult viability, which is measured as the proportion of eggs developing successfully into adults, we used a logistic regression in a generalised linear model. We used the same model constructs and comparisons as for the starvation resistance (see above) but compared models using likelihood ratio tests to obtain *P*-values. Plots were produced using R packages ‘ggplot2’ v.3.3.6 (https://ggplot2.tidyverse.org; Wickham, 2016), ‘patchwork’ v.1.1.2 (https://CRAN.R-project.org/package=patchwork) and ‘ggbreak’ v.0.1.1 (Xu et al., 2021). All assumptions of the parametric analyses were fulfilled, and we detected no over- or under-dispersion in the logistic regression.

#### Gene expression data

Differential gene expression analysis was conducted using gene by gene comparisons in the R package ‘limma’ v.3.50.3 (Ritchie et al., 2015). This approach produces an estimate of fold-change and a *P*-value corrected for multiple testing for each gene when comparing two treatments. We focused our analysis on three questions, each of which could be investigated by several treatment comparisons. (1) To test the consistency in gene expression across the experimental generations, we compared gene expression levels among the Rich diet treatments in F0, F1 and F2, focusing on flies with parents developed on the Rich diet (F0_rich_ versus F1_rich_, F1_rich_ versus F2_rich_, F0_rich_ versus F2_rich_). All else being equal we expected no differences in expression among these identical treatments. (2) Next, we investigated the impact of nutritional stress on gene expression by comparing expression levels between the Rich diet and the Poor diet treatment within F0, F1 and F2, focusing only on flies with parents developed on the Rich diet (F0_rich_ versus F0_poor_, F1_rich_ versus F1_poor_, F2_rich_ versus F2_poor_). (3) Finally, we tested whether the diet quality of parents impacted the gene expression of their offspring. We first focused on offspring developed on the Poor diet as we were particularly interested in the priming of offspring induced by reductions in nutritional quality [F1_poor_ (mothers at rich) versus F1_poor_ (mothers at poor), F2_poor_ (grandmothers at rich) versus F2_poor_ (grandmothers at poor)]. Next, we constructed models in the limma R-package containing interactions between F0 and F1, to investigate whether any genes were differentially expressed as a result of maternal diet. *P*-values were adjusted using the false discovery rate method (FDR).

## RESULTS

### Flies exposed to a gradient of nutritional stress in F0

**Fig. 2.**
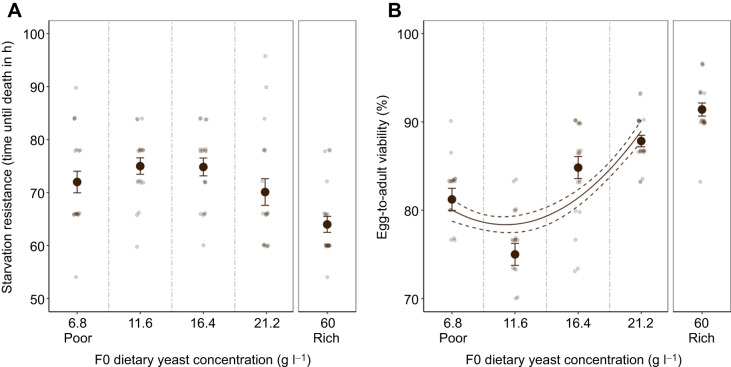
**Reduction in available yeast during development reduces egg-to-adult viability but not adult starvation resistance.** (A) Starvation resistance (time to death in hours) and (B) egg-to-adult viability of F0 *D. melanogaster* that developed over a range of nutritional stress (means±s.e.m.). Small grey points show individual time until death (number of flies per treatment at each dietary yeast concentration: 6.8 g l^−1^ *n*=20, 11.6 g l^−1^ *n*=18, 16.4 g l^−1^ *n*=19, 21.2 g l^−1^ *n*=19, 60 g l^−1^ *n*=21) and egg-to-adult viability per vial (number of vials per treatment: 6.8 g l^−1^ *N*=19, 11.6 g l^−1^ *N*=20, 16.4 g l^−1^ *N*=20, 21.2 g l^−1^ *N*=20, 60 g l^−1^ *N*=19) jittered ±0.25 across the *x*- and *y*-axis for better visualisation. Where a significant difference was found, the fitted lines represent the predicted response based on a linear model with yeast amount (continuous) as the sole predictor variable (solid line) and standard error (dashed lines). Results from flies exposed to the Rich diet are presented for visual comparison, but were not included in the analyses because of the increased complexity of the medium (Table 1). The survival curve for the starvation resistance assay can be found in Fig. S1.

Before evaluating the presence of generational effects, we quantified the direct impact of the nutritional gradient. We detected no impact of nutrition levels on adult starvation resistance (*F*_1,75_=0.36, *P*=0.55; Table S1; Fig. 2A), whereas egg-to-adult viability was progressively reduced for diets with lower amounts of yeast (χ^2^=28.68, *P*<0.001; Table S1; Fig. 2B), showing that we were successful in exposing flies to a gradient of nutritional stress.

### Nutritional stress tolerance depends on the maternal environment

**Fig. 3.**
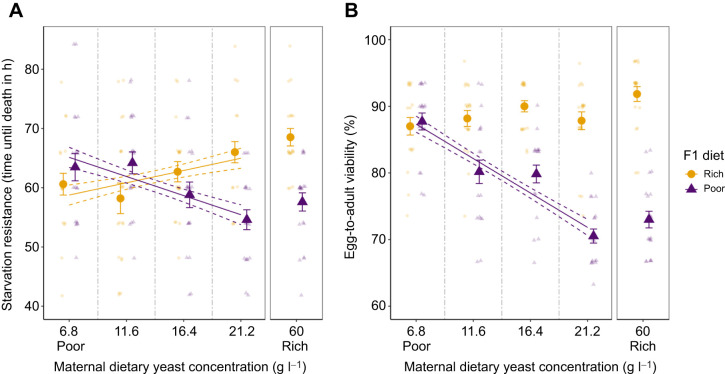
**Performance of flies exposed to nutritional stress depends on the nutritional environment of their parents.** (A) Starvation resistance and (B) egg-to-adult viability of flies with mothers that developed over a range of nutritional stress (means±s.e.m.). Flies were developed on either the Rich diet (yellow circles) or the Poor diet (purple triangles). Small opaque points show individual time until death (number of flies per treatment: Rich: 6.8 g l^−1^ *n*=20, 11.6 g l^−1^ *n*=20, 16.4 g l^−1^ *n*=20, 21.2 g l^−1^ *n*=20, 60 g l^−1^ *n*=19; Poor: 6.8 g l^−1^ *n*=19, 11.6 g l^−1^ *n*=20, 16.4 g l^−1^ *n*=20, 21.2 g l^−1^ *n*=20, 60 g l^−1^ *n*=20) and egg-to-adult viability per vial (number of vials per treatment: Rich: 6.8 g l^−1^ *N*=20, 11.6 g l^−1^ *N*=20, 16.4 g l^−1^ *N*=20, 21.2 g l^−1^ *N*=20, 60 g l^−1^ *N*=20; Poor: 6.8 g l^−1^ *N*=19, 11.6 g l^−1^ *N*=20, 16.4 g l^−1^ *N*=20, 21.2 g l^−1^ *N*=20, 60 g l^−1^ *N*=20) jittered +0.75 for the Poor diet (purple) and −0.75 for the Rich diet (yellow) across the *x*-axis and ±0.25 across the *y*-axis for better visualisation. Where a significant difference was found, the fitted lines represent the predicted response based on a linear model where the maternal dietary yeast (F0 diet) is a continuous predictor variable (solid line) and standard error (dashed lines). Survival curves for the starvation resistance assay can be found in Fig. S2A (flies developed on the Rich diet) and Fig. S2B (flies developed on the Poor diet).

The nutritional effects also transcended to the next generation, affecting performance of flies on the Poor diet and the Rich diet in opposing ways (F0 by F1 interaction: *F*_3,156_=17.16, *P*<0.001; Table S1; Fig. 3A). Starvation resistance of the F1 flies developed on the Poor diet decreased as the amount of yeast in their parents' diet increased (*F*_1,77_=12.76, *P*<0.001; Fig. 3A). In contrast, the starvation resistance of the F1 flies developed on the Rich diet increased when the amount of yeast in their parents' diet increased (*F*_1,78_=5.24, *P*<0.05; Fig. 3A). Consequently, flies with parents developing at lower nutritional stress suffered reductions in starvation resistance when developing at the Poor diet compared with the Rich diet, whereas flies with parents developing at high nutritional stress were unaffected by the transition from the Rich to the Poor diet.

This ability of parents experiencing stressful diets to prime their progeny to cope with similar stressful diets was supported by analyses of egg-to-adult viability. Here, we also found a significant interaction between the F0 and F1 diets (χ^2^=18.35, *P*<0.001; Table S1; Fig. 3B), which was caused by a reduction in egg-to-adult viability on the Poor diet, as the yeast concentration of their parents' diet increased (χ^2^=47.55, *P*<0.001; Table S1; Fig. 3B). Hence, while the reduction in egg-to-adult viability on the Poor diet was substantial for offspring of parents exposed to nutritious diets (e.g. at 21.2 g l^−1^; Fig. 3B), no effect of the Poor diet was observed in flies of parents that were themselves reared on the Poor diet (at 6.8 g l^−1^; Fig. 3B).

### One generation with rich nutrition reduces generational priming for poor nutrition

**Fig. 4.**
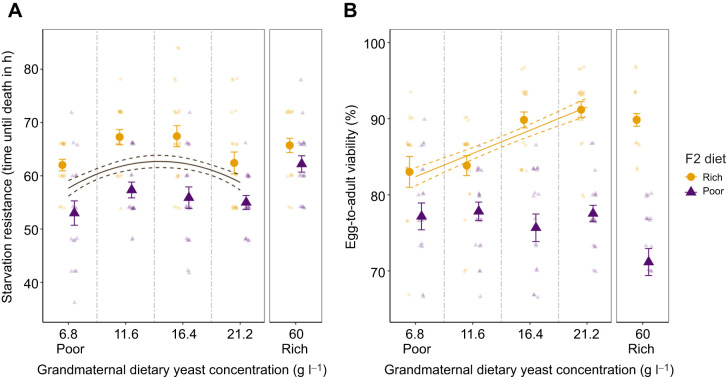
**One generation under optimal nutrition reduces maternal effects.** (A) Starvation resistance and (B) egg-to-adult viability of *D. melanogaster* flies with grandmothers (F0) that developed over a range of nutritional stress (means±s.e.m.). These F2 flies were developed on either the Rich diet (yellow circles) or the Poor diet (purple triangles). Small opaque points show individual time until death (number of flies per treatment: Rich: 6.8 g l^−1^ *n*=18, 11.6 g l^−1^ *n*=19, 16.4 g l^−1^ *n*=21, 21.2 g l^−1^ *n*=20, 60 g l^−1^ *n*=20; Poor: 6.8 g l^−1^ *n*=18, 11.6 g l^−1^ *n*=18, 16.4 g l^−1^ *n*=19, 21.2 g l^−1^ *n*=18, 60 g l^−1^ *n*=19) and egg-to-adult viability per vial (number of vials per treatment: Rich: 6.8 g l^−1^ *N*=20, 11.6 g l^−1^ *N*=20, 16.4 g l^−1^ *N*=20, 21.2 g l^−1^ *N*=20, 60 g l^−1^ *N*=20; Poor: 6.8 g l^−1^ *N*=20, 11.6 g l^−1^ *N*=20, 16.4 g l^−1^ *N*=20, 21.2 g l^−1^ *N*=19, 60 g l^−1^ *N*=20) jittered +0.75 for the Poor diet (purple) and −0.75 for the Rich diet (yellow) across the *x*-axis and ±0.25 across the *y*-axis for better visualisation. The mothers (F1) of the tested flies were exposed to the Rich diet. Where a significant difference was found, the fitted lines represent the predicted response based on the linear model where grandmaternal dietary yeast (F0 diet) is a continuous predictor variable (solid line) and standard error (dashed lines). Survival curves for the starvation resistance assay can be found in Fig. S3A (flies developed on the Rich diet) and Fig. S3B (flies developed on the Poor diet).

The impact of the nutritional stress in F0 flies on starvation resistance in F1 flies (Fig. 3A) disappeared in F2 flies after one generation on the Rich diet (F0 by F2 interaction: *F*_1,147_=5.67, *P*=0.57; Table S1; Fig. 4A). Starvation resistance across all flies in the F2 followed a quadratic relationship with the F0 diet (*F*_2,150_=3.82, *P*<0.05), where resistance was highest for the intermediate yeast concentrations. The distinct differences between Rich and Poor diets detected in the F1 flies (Fig. 3A) were therefore not re-found here. This indicates that priming effects detected in F1 were highly transient, so that primarily the diet of the parents, not the grandparents, determined the ability of flies to cope with nutritional stress.

This finding was also supported by analysis of egg-to-adult viability (Fig. 4B). Here, we found a significant interaction between the F0 and F2 diets (χ^2^=15.77, *P*<0.001; Table S1), but this interaction was not caused by generational effects on the egg-to-adult viability of flies on the Poor diet (χ^2^=0.02, *P*=0.88; Table S1). Egg-to-adult viability of flies on the Poor diet was therefore unaffected by their grandparents' diet, supporting that the priming to Poor diets is transient. We did however find generational effects in flies developed on the Rich diet, which had reduced egg-to-adult viability with increasing nutritional stress (χ^2^=24.82, *P*<0.001; Table S1).

### Limited role of constitutive gene expression in transmitting generational effects

**Fig. 5.**
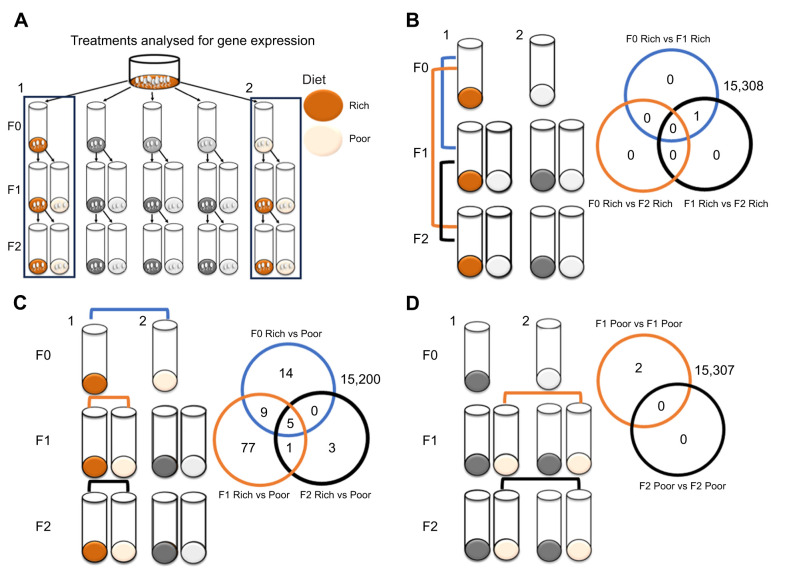
**Nutritional background has a limited impact on gene expression.** (A) Effects of nutritional quality on gene expression, and its persistence across generations were investigated by focusing on flies from Rich and Poor diet treatments in F0 and their progeny. We performed three different analyses. (B) First, we tested for a temporal change in gene expression across generations in flies from identical nutritional treatments that would bias the results of other contrasts. This was done by three different treatment contrasts (blue, orange and black), with the number of significant differentially expressed genes for each contrast shown in the Venn diagram. (C) Second, we identified genes that were differentially expressed as a result of development under poor versus rich nutrition. (D) Finally, we tested for maternal and grandmaternal effects on gene expression. Numbers outside the Venn diagram show the number of genes not significantly differentially expressed.

We first established whether there were any temporal changes in gene expression across generations as a result of unwanted experimental artefacts that could bias the results of our treatment comparisons. This was possible to test as all three generations of flies (F0, F1 and F2) included a treatment where the focal flies and their previous generations had developed on the Rich diet (Fig. 5A,B). Out of 15,309 genes, we found just a single gene (*Larval serum protein 2*; Fig. 5B) that had a significant difference in expression across generations in flies from the Rich diet. This is an important validation of our experimental and analytical approach, showing high consistency in the environmental responses to the same diet across generations. We can therefore be confident that any identified gene expression differences are the result of diet exposure rather than uncontrolled environmental variation across generations.

We then compared expression levels of flies experiencing nutritionally rich diets in F0, F1 and F2 with those of flies developed on the Poor diet across three generations. This allowed us to establish a baseline of the genes that are differentially expressed as a result of development under poor nutrition for one generation. The differentially expressed genes are probably involved in generational responses to nutritionally poor diets (Fig. 5C). Out of 15,309 genes, 28 were significantly differentially expressed in the F0, 92 in the F1 and 9 in the F2 (Fig. 5C; Table S2). Of the 109 total genes found to be significant in at least one of these generations (Fig. 5C), 63 genes were downregulated and 28 genes where upregulated under poor nutrition in all generations (Table S2, Fig. S4). This gene set of 91 genes with consistent direction of gene expression change across diet quality was used below as a putative candidate gene set for the maternal priming.

When testing for maternal effects in the constitutive expression levels, we compared flies developed on the Poor diet in the F1 with the maternal generation developed on either the Poor or Rich diet (Fig. 5D). No genes were significant for the interaction between the maternal diet and offspring diet, but we found two genes with higher expression in flies whose mothers had developed on the Rich diet compared with the Poor diet (Fig. 5D). Unfortunately, neither of the two genes was annotated: *CG9568* gene product from transcript CG9568-RA and *CG5399* gene product from transcript CG5399-RB (Table S2). To test for similar gene expression responses between direct responses to nutritional stress and maternal priming to nutritional stress, we tested whether any of the 91 putative candidate genes were in the top 100 most significant genes in the analysis for maternal effects on gene expression. We found five genes that overlapped between these two gene sets, which included the two genes that were significant for maternal effects, *thetaTrypsin* gene involved in proteolysis and expressed during digestion in the midgut, *CG43774* gene product from transcript CG43774 and *CG14500* gene product from transcript CG14500 involved in carbohydrate binding. To test for grandmaternal effects on gene expression, we focused on flies developed on the Poor diet in F2, with grandmothers developed on either the Poor or Rich diet (Fig. 5D). We found no significantly differentially expressed genes related to grandmaternal effects (Fig. 5D). This is in line with the lack of impact of grandmaternal diet on performance (egg-to-adult viability and starvation tolerance) on the Poor diet (Fig. 4).

## DISCUSSION

Using a serial dilution of nutrients, we demonstrated that reductions in nutrient availability during development of one generation primes the viability of juveniles and starvation resistance of adults in the subsequent generation when exposed to similar nutritional challenges to those experienced by their parents. This is probably an adaptive mechanism evolved to cope with environments where the nutritional environments vary in space or time (Mousseau and Fox, 1998). Based on our gradient of nutritional stress, the results indicate that priming of the next generation is not a threshold effect where it is activated at a particular stress level. Instead, performance in both egg-to-adult viability and adult starvation resistance under poor nutrition appears to continuously increase as a function of the nutritional stress experienced in the previous generation (Fig. 3).

Increasing starvation resistance under poor nutrition may have costs for other life-history traits, making the distinction of adaptive and maladaptive responses challenging. Vijendravarma et al. (2010) showed in *D. melanogaster* that offspring of mothers developed on a nutritionally poor diet developed quicker but became smaller on a poor diet compared with offspring of mothers developed on a nutritionally rich diet, and Deas et al. (2019) found trade-offs between ovariole number and egg volume in *D. melanogaster* also developing under nutritional stress. Indeed, long-term exposure to lower nutritional quality food may not always be ‘positive’. Kawecki et al. (2021) found that after 150 generations of adaptation to a poor larval diet, there was a genetically based decrease in starvation resistance in adults. Similarly, it is possible that correlated responses can explain findings in our experiment in traits such as adult fat content (Rehman and Varghese, 2021), body mass or developmental time.

The fitness consequences of correlated trait responses probably depend on the phenotypic match with environmental stressors. Successful priming requires that the nutritional environment is predictive across generations, a criterion that is more likely to be met in animals with very short generation time and little migration. In line with this, the spatially restricted springtail *Orchesella cincta* also shows evidence of nutritional priming (Zizzari et al., 2016). Unfortunately, the design of two generations and two nutritional treatments did not allow the scale of these responses and their persistence across generations to be investigated. We found that the priming to nutritional challenges was transient and disappeared after just one additional generation of development on the Rich diet. Interestingly, generational effects on life-history traits, such as reproduction, have been reported to be more persistent (Deas et al., 2019). It is therefore possible that F2 flies in our study were still impacted by the nutritional stress of their grandparents, but without a manifestation on their performance when reared under poor nutrition. Alternatively, the discrepancy in persistence between studies may be due to variation in nutritional stress levels (which was not evaluated in the study by Deas et al., 2019). We found the priming to poor nutrition to be transient, but there was some evidence of a reduced performance of flies on the Rich diet if their grandparents experienced poor nutrition and only in egg-to-adult viability (Fig. 3). Such negative generational effects have also been identified in beetles exposed to poor diets (Kyneb and Toft, 2006) and in lizards reproducing at an old age (Bleu et al., 2021).

A substantial part of the variation in findings across studies is probably due to the use of different sexes. Generally, studies investigating the impact of diet across one or more generations have found sex-specific responses (Dew-Budd et al., 2016; Davies et al., 2018; Duxbury and Chapman, 2020; McDonald et al., 2021; Krittika and Yadav, 2022; Lushchak et al., 2023). For example, when *D. melanogaster* flies are exposed to nutrient deficiencies during their larval stage, only males adjust their nutritional intake as adults, causing sex dimorphism in starvation resistance (Davies et al., 2018). There are also substantial differences in the effect of maternal diets and paternal diets on the performance of female and male progeny (Dew-Budd et al., 2016). When testing for offspring priming, studying maternal and paternal effects therefore warrants independent investigations (Lecomte et al., 2013; Dew-Budd et al., 2016). These sexual dimorphisms are likely to arise from sex differences in physiology and metabolism, partly as a result of the females' egg production demands (Partridge et al., 2005; Sieber and Spradling, 2015; Jang and Lee, 2015; Millington and Rideout, 2018). Mechanistic insight into the role of metabolism in starvation resistance stems from studies of lines selected for starvation tolerance of *D. melanogaster* (Brown et al., 2019), which found that selection lines showed delayed development and reduced metabolic rate of both larvae and adults. Additionally, behavioural responses such as increased duration of sleep and reduced feeding activity have also been described (Masek et al., 2014; Slocumb et al., 2015). While the exact genetic pathways are not always understood, these mechanisms can be linked to the need to conserve energy under restricted access to food. Interestingly, a generational effect on metabolism is described in *Drosophila*, supporting that this mechanism might be involved in explaining our results (Buescher et al., 2013).

We found little evidence for nutritional priming being encoded in the constitutive gene expression of adults, with only five (two significant) genes detected. This is not because responses to nutritional stress exclude constitutive gene expression, as we found more than 100 genes being differentially expressed between flies on the Rich and Poor diet. Instead, the mechanism underlying generational effects appears to be priming the offspring to initiate an adaptive plastic response only when exposed to nutritionally poor diets, or priming constitutive changes during the larval stage improving adult performance under poor dietary conditions. When we compared the >100 differentially expressed genes between the Rich and Poor diet treatments with studies identifying genes responding to selection for starvation resistance (Hardy et al., 2018) or genes involved in the starvation stress response (Moskalev et al., 2015), we found no overlap of the identified genes. This supports that the increase in starvation resistance during generational priming is activated by phenotypic plasticity. Such changes in phenotypic plasticity may also reduce the potential costs of generational priming by only investing in a specialisation to poor nutrition when the likelihood of experiencing such conditions is high. Future work should target temporal changes in gene expression during nutritional stress across life stages to detect key genes of interest and pinpoint mechanisms underlying generational priming. This is needed if we are to understand the potential costs of generational priming and thereby the situations in which such priming is an adaptive stable strategy.

## Supplementary Material

10.1242/jexbio.247972_sup1 Supplementary information

Table S1. Full results of the sequential model reduction for starvation resistance and egg-to-adult viability. Bold denotes significant p-value.

Table S2. Gene lists for the rich vs poor developmental diet comparisons across the three generations: Significantly differentially expressed genes, genes that are upregulated in flies developed on the poor diet and genes that are downregulated in flies developed on the poor diet.
