# Supplementary material for: Nutritional stress in larvae induces adaptive responses that transcend generations in males of a model insect
Source: J Exp Biol. 2025 Jan 17;228(2):jeb247972. doi: 10.1242/jeb.247972 (PMC11832117; doi:10.1242/jeb.247972)
Supplement: Supplementary information [file jexbio-228-247972-s1.pdf]

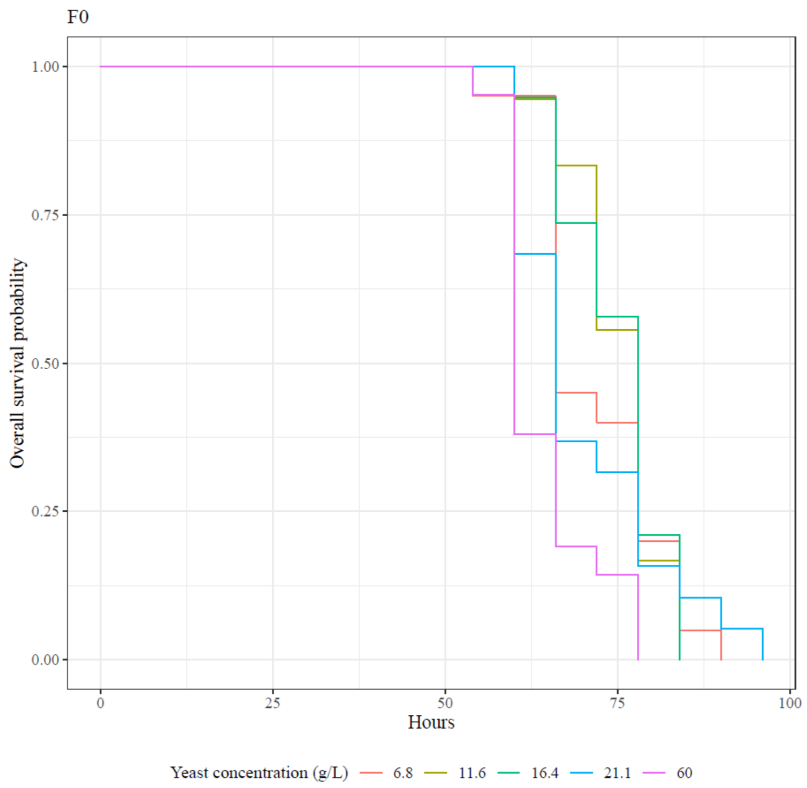


**Fig. S1.** Survival curves of **F0** flies subjected to the starvation assay.

A.

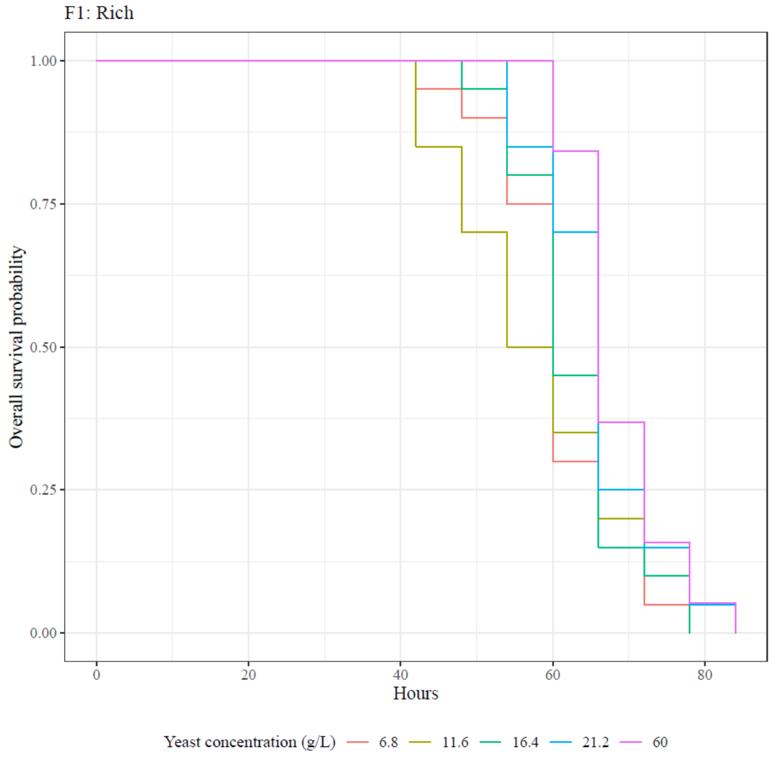


B.

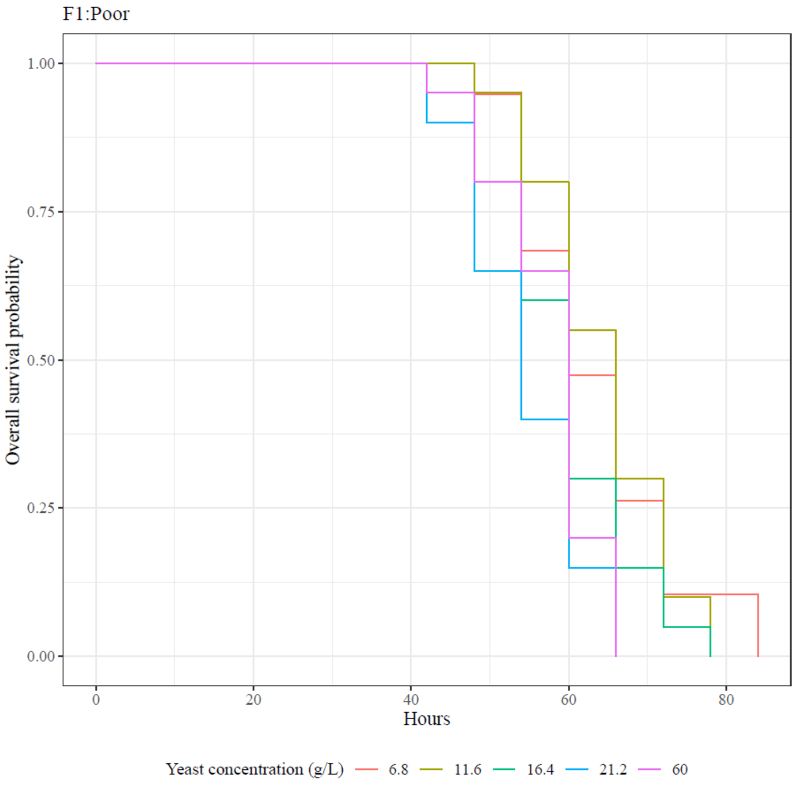


**Fig. S2.** Survival curves of **F1** flies subjected to the starvation assay. A) F1 flies developed on the rich diet. B) F1 flies developed on the poor diet.

Journal of Experimental Biology • Supplementary information

A.

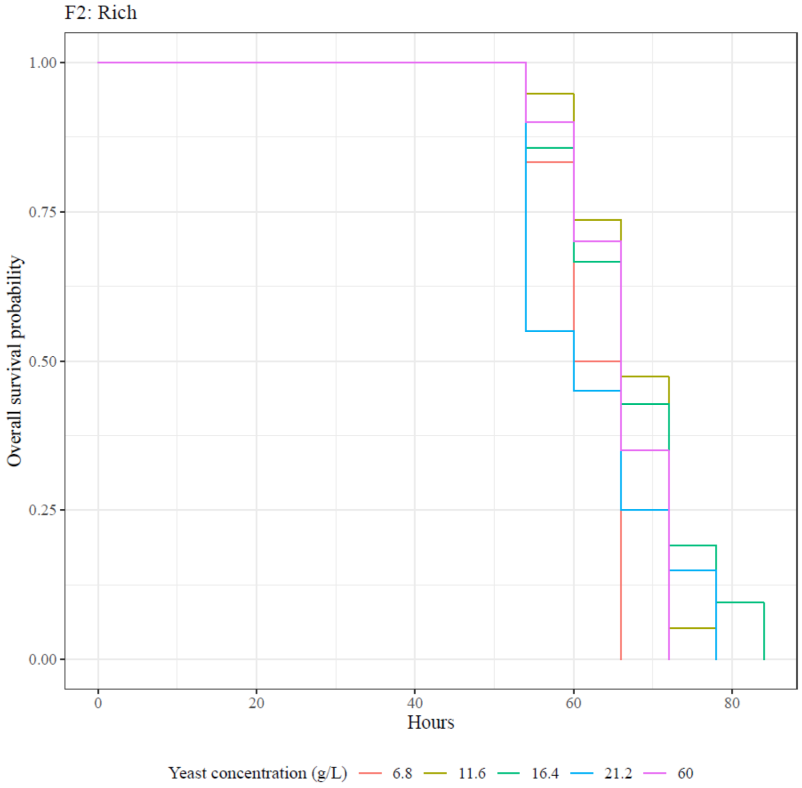


B.

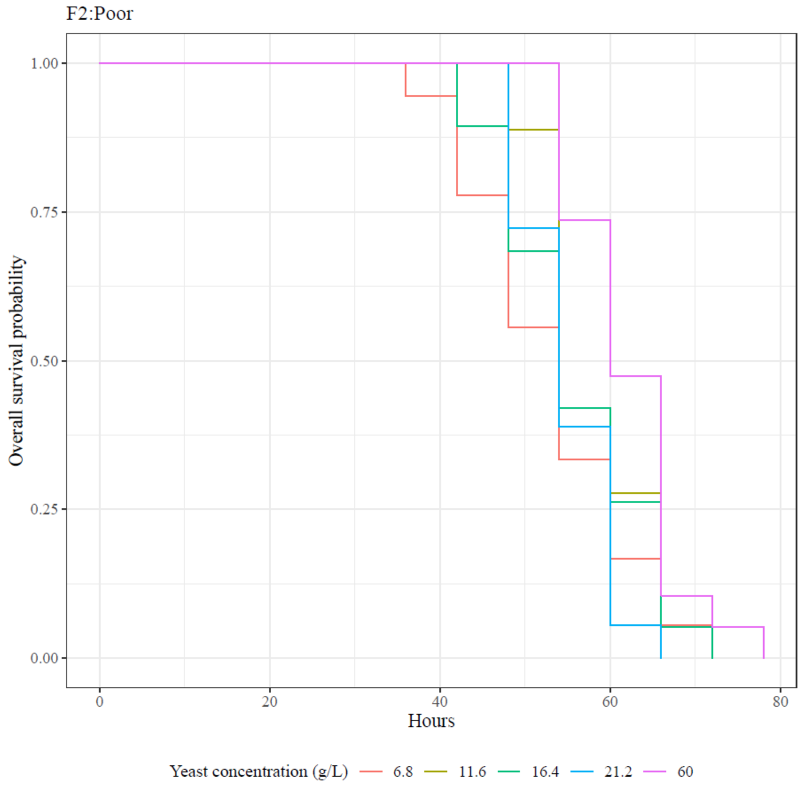


**Fig. S3.** Survival curves of **F2** flies subjected to the starvation assay. A) F2 flies developed on the rich diet. B) F2 flies developed on the poor diet.

Journal of Experimental Biology • Supplementary information

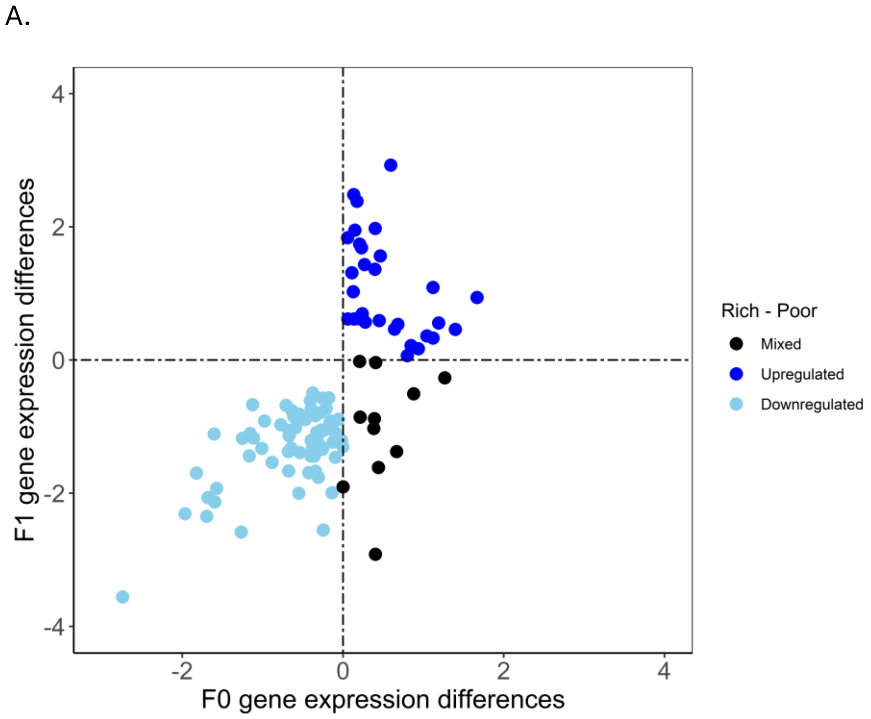
A.
4
2
0
-2
-4
F1 gene expression differences
-2
0
2
4
F0 gene expression differences
Rich - Poor
Mixed
Upregulated
Downregulated

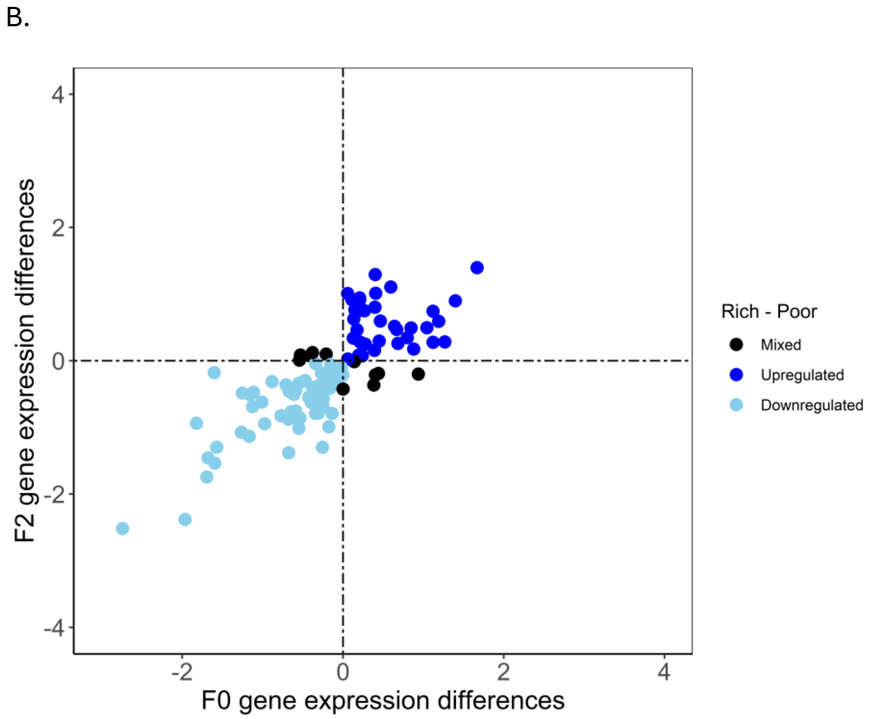
B.
4
2
0
-2
-4
F2 gene expression differences
-2
0
2
4
F0 gene expression differences
Rich - Poor
Mixed
Upregulated
Downregulated

C.

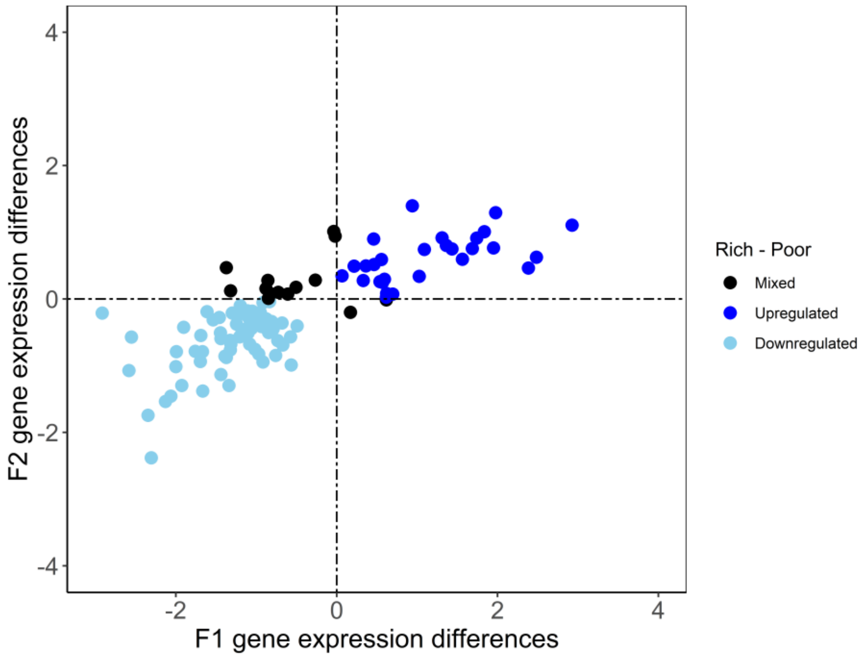


**Fig. S4.** Correlation plots of 109 genes that were found to be significantly different between flies developed on rich diets vs flies developed on poor diets across the three generations. Axis shows the differential gene expression levels (Log fold change) within each generation. If diagonal, the genes therefor show similar expression patterns in each generation comparison. A) F0 vs F1, B) F0 vs F2 and C) F1 vs F2.

**Table S1.** Full results of the sequential model reduction for starvation resistance and egg-to-adult viability. Bold denotes significant p-value.

Available for download at
https://journals.biologists.com/jeb/article-lookup/doi/10.1242/jeb.247972#supplementary-data

**Table S2.** Gene lists for the rich vs poor developmental diet comparisons across the three generations: Significantly differentially expressed genes, genes that are upregulated in flies developed on the poor diet and genes that are downregulated in flies developed on the poor diet.

Available for download at
https://journals.biologists.com/jeb/article-lookup/doi/10.1242/jeb.247972#supplementary-data
